# Study on the Expansion Law of Dry Shrinkage Cracks in Silty Clay

**DOI:** 10.3390/ma15113941

**Published:** 2022-06-01

**Authors:** Jianwei Yue, Peng Li, Limin Zhao, Xuanjia Huang, Xiangchun Xu, Zifa Wang

**Affiliations:** 1 School of Civil Architecture, Henan University, Kaifeng 475004, China; yjwchn@126.com (J.Y.); lpeng0114@163.com (P.L.); hxj_henu@126.com (X.H.); xxc_geo@foxmail.com (X.X.); zifa@iem.ac.cn (Z.W.); 2 Kaifeng Key Laboratory of Restoration and Safety Evaluation of Immovable Cultural Relics, Kaifeng 475004, China

**Keywords:** earthen sites, drying shrinkage, crack morphology, cracking law

## Abstract

Fracture characteristics are the basis of silty clay fracture research, and its quantitative description is helpful to explore the engineering properties of silty clay. The silty clay samples with different moisture contents and different aspect ratios were prepared by the controlled variable method for the drying shrinkage test. The crack image processing and crack feature extraction were performed by PS and IPP software, and the relationship between the crack propagation characteristic parameters and the change in humidity and sample moisture content during drying shrinkage were analyzed to explore the crack development law. The results show that under the continuous action of the environment, cracks were generated at the initial high temperature (46 °C). When the temperature changed from a high temperature (46 °C) to a low temperature (27 °C), the moisture content decreased faster, and the crack developed rapidly. Later, from low temperature (12 °C) to high temperature (46 °C), the water content and crack parameters remained basically unchanged; the cracks of the samples with a high moisture content appeared early, developed rapidly, and damaged seriously. When the aspect ratio was 6:1, the crack developed vertically, and when the aspect ratio was less than 6:1, the secondary crack was about 90° from the upper crack.

## 1. Introduction

**Figure 1:**
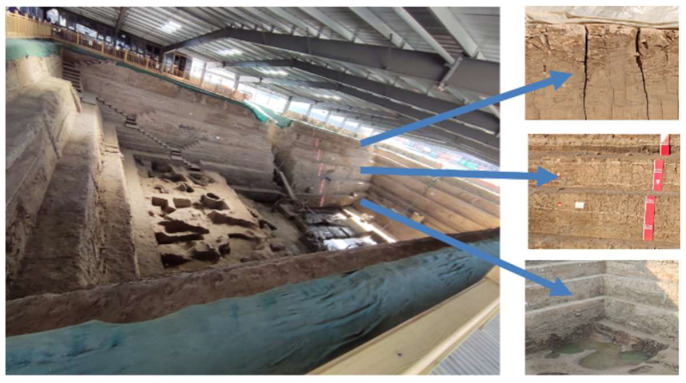
Shrinkage and deterioration of the Zhouqiao site in Kaifeng.

Historically, the middle and lower reaches of the Yellow River were the center of Chinese activities for a long period of time, making many earthen sites in the middle and lower reaches of the Yellow River [1]. The composition of soil sites in the middle and lower reaches of the Yellow River is mainly powdered clay, because powdered clay has the characteristics of fine particles, low strength, poor water stability, etc. Under the repeated action of the external environment, many soil sites have produced cracking, spalling and chalking, and other diseases on the surface [2,3,4,5]. This experiment explored the dry shrinkage fracture expansion pattern of powdered clay using the Zhouqiao site as an example (Figure 1). The sprouting and expansion of these initial fissures not only destabilize the site proper, but also provide a channel for moisture migration, exacerbating soil shrinkage and weakening the physical and mechanical properties. At present, dry shrinkage cracking has occurred in the surface layer of many soil sites in China, and it has become an urgent engineering problem to explore the fracture expansion law of powdered clay under dry shrinkage conditions for preventive conservation work.

As earthen sites have been excavated in recent years, many of them have undergone dry shrinkage and cracking during excavation. Scholars at home and abroad have studied the cracking phenomenon of earthen sites and found that drying shrinkage is the most important factor affecting the cracking of earthen sites. During the actual excavation of the soil site, the water in the soil will continuously evaporate in the air, resulting in changes in the surface tension between the soil particles, and eventually the surface layer of the soil site causes different degrees of dry shrinkage and deterioration [6,7]. Influenced by the soil sample’s own characteristics, the deterioration of the site soil needs to be studied by several factors, and only considering a single factor cannot well derive the pattern of cracking of the site soil, which will cause differences between the test results and the actual fissure development. For example, considering only a single thickness will not capture the effect of moisture gradients in the depth direction on the development of fissures in powdery clays. There is also a soil sample size effect on soil sample cracking, where the area of disturbance caused by different excavation areas (sizes) during site excavation varies, which in turn affects the stability of the soil sample and causes cracking of the soil sample. Therefore, it is necessary to investigate the deterioration mechanism of site soils from multiple perspectives and factors. Considering the problems of complex and changing external environmental conditions, high test costs and low-test efficiency when conducting field tests, and combined with image processing technology, the progress of soil sample cracking can be accurately grasped, which provides technical support for a reasonable analysis of the fissure expansion pattern [8,9,10].

Under the repeated action of the external environment, diseases such as cracks, denudation, and powdering appear on the surface. At present, much of the research on soil water loss and cracking mainly focuses on the influence of a single factor on soil cracking or the soil’s dry–wet cycle and numerical simulation. The test conditions are ideal and difficult to combine with real working conditions. This article starts from reality, refers to and simulates changes in the field environment, and is closer to the actual working conditions [10,11]. In this paper, the drying shrinkage test was carried out by the controlled variable method, and the influencing factors such as temperature, humidity, initial moisture content of the sample, and different aspect ratios are considered. Through image acquisition and processing technology, Photoshop (ADOBE SYSTEMS INCORPORATED, San Jose, CA, USA) and Image-Pro Plus 6.0(MEDIA CYBERNETICS, Rockville, MD, USA) software were used to binarize and denoise the image to study the effect of shrinkage on the crack area, crack direction, and crack angle of the field soil samples. The research results provide a certain basis for the preventive protection of the site soil.

## 2. Experimental Materials and Protocols

### 2.1. Site Soil

The state bridge site is a newly excavated site, and the bottom elevation is lower than the horizon, although there is a roof in its upper part to avoid rain and snow damage, but due to the exposure to the open environment, the long-term action of the natural external camp force has led to the comprehensive development of a variety of diseases, which are completely different from the existing building under the action of the environment with weather resistance characteristics. Therefore, it is of practical significance to explore the deterioration pattern and mechanism of the site soil.

The soil samples used for the test were taken from the site soil of the Kaifeng Zhouqiao site, which was yellow in color with a good cohesiveness and plasticity. The retrieved soil samples were subjected to geotechnical tests in accordance with the Standard for Geotechnical Test Methods (GB/T50123-2019), and the basic physical properties of the site soil are shown in Table 1.

### 2.2. Dry Shrinkage Test

In order to realistically simulate the damage of the soil at the Kaifeng State Bridge site under dry shrinkage conditions, the experimental temperature and humidity were selected with reference to the historical weather in Kaifeng [12,13]. That is, the maximum temperature is 46 °C, the minimum temperature is 12 °C, and the average temperature is 27 °C; the maximum humidity is 95%, the minimum humidity is 30%, and the average humidity is 67%. The water content was selected as 38% of the liquid limit, 21% of the plastic limit, and 30% of the average value of the liquid–plastic limit, respectively, and the specific experimental scheme is shown in Table 2.

The specimen preparation process was as follows. After drying the site soil in the oven and passing through a 2 mm sieve, the sample was prepared by weighing the corresponding mass of soil and water, mixing the soil and water evenly, and trying to pinch the larger clusters apart, and then the soil sample was sealed for 24 h using cling film. Due to the different stress changes in the dry shrinkage process for specimens with different aspect ratios [14], the soil samples were mixed with different aspect ratios in combination with the excavation step ratio of the state bridge site, and the different aspect ratio specimens were taken as 12:1, 6:1, 3:1, and 1.5:1. The height was uniformly taken as 2 cm. Finally, the length × width × height of the specimens was 60 cm × 5 cm × 2 cm, 60 cm × 10 cm × 2 cm, 30 cm × 10 cm × 2 cm, and 30 cm × 20 cm × 2 cm, respectively. The samples under different conditions sit on three sets of the same parallel samples. The data processing variances of the crack area, crack orientation and crack angle are shown in Table 3, Table 4 and Table 5.

The dry shrinkage test procedure was as follows: ① prepare specimens, take photos and weigh them according to Table 2; ② weigh and take photos of the specimens put into the environmental simulation box every 1 h; ③ experiment in each group until the moisture content no longer changes and stops. In order to explore the evolution of the crack network of soil samples caused by dry shrinkage, the surface fracture structure of soil samples under dynamic dry shrinkage condition was monitored.

**Figure 2:**
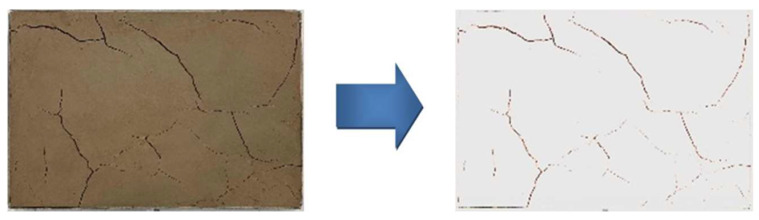
Image processing process.

During the experiment, the camera equipment and specimen mold positioning slots were fixed to ensure that the specimens were fixed in the same position each time the pictures were taken. Photoshop and Image-Pro Plus 6.0 software were used to binarize and noise-reduce the captured images in order to analyze a series of indicators of crack area, the ratio between the long and short axes of the specimen’s crack closure pattern, and the change in the sum of angular development in the crack development pattern. The effect of dry shrinkage on the fracture area, fracture orientation, and fracture angle of the site soil samples was investigated comprehensively. The image processing flow is shown in Figure 2.

## 3. Analysis of Experimental Results

In this paper, the method of extracting fracture characteristic parameters was improved based on the studies of scholars Zhao Fang [15,16], Liu Chun [17,18], and Liu Chunshang [19] and used to collect the characteristic parameters of fracture development in soil samples under dry shrinkage conditions and to statistically analyze the effects of different experimental variables on fracture area, fracture orientation, and fracture angle.

### 3.1. Analysis of Macroscopic Experimental Cracking Image Results

Macroscopically, evaporation is an important prerequisite for soil shrinkage cracking. During the drying process, the soil loses water and evaporates, and suction is generated inside the soil due to capillary action. With the evaporation and drying of water in the soil to a certain extent under the continuous action of the environment, there will also be weak points in some local areas. Cracking will occur. In the stage of rapid decrease in moisture content, the corresponding sample begins to crack to form cracks, and the water is lost from the surface to the inside. With the evaporation of water, it gradually transitions to the deceleration stage. At this time, the increase in the crack parameters gradually slows down until it reaches the residual stage, the water evaporation rate is reduced to 0, and the cracks no longer increase [20,21,22].

Microscopically, soil cracking is a typical form of tensile failure and is the result of stress. During the drying process of the soil, matrix suction will be generated. When the tensile stress field caused by the matrix suction exceeds the tensile strength of the soil itself, cracks will form, and the tip of the crack will form a new tensile stress field concentration point, resulting in cracks. There will be continuous development under tip traction. Therefore, the matrix suction and the tensile strength of the soil are two key mechanical indicators restricting the development of cracks. It can be seen from the figure that the matrix suction of silty clay increased with the decrease in saturation, and the tensile strength decreased with it. As the test proceeded, the matrix suction inside the sample exceeded the tensile strength of the sample. When the sample was cracked, the moisture content decreased continuously, and the suction force and tensile stress of the matrix continued to change, until the water evaporation rate decreased to 0 in the residual stage [21].

**Figure 3:**
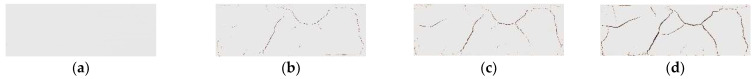
Cracking of specimens with a size of 30 cm × 10 cm × 2 cm. (**a**) Initial stage; (**b**) crack development stage; (**c**) crack development stage; (**d**) final crack morphology.

**Figure 4:**
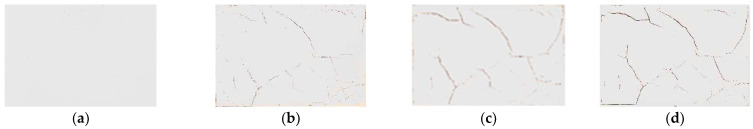
Cracking of specimens with a size of 30 cm × 20 cm × 2 cm. (**a**) initial stage; (**b**) crack development stage; (**c**) crack development stage; (**d**) final crack morphology.

**Figure 5:**
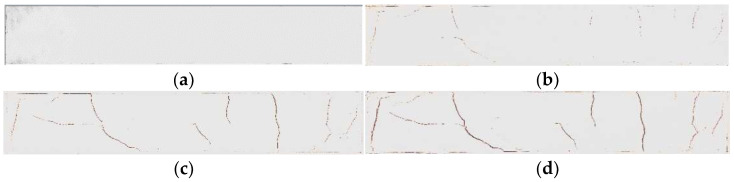
Cracking of specimens with a size of 60 cm × 10 cm × 2 cm. (**a**) initial stage; (**b**) crack development stage; (**c**) crack development stage; (**d**) final crack morphology.

**Figure 6:**
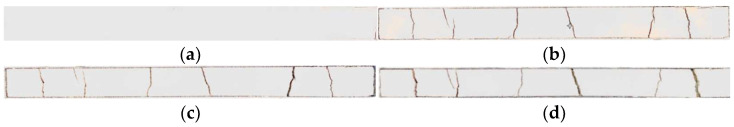
Cracking of specimens with a size of 60 cm × 5 cm × 2 cm. (**a**) initial stage; (**b**) crack development stage; (**c**) crack development stage; (**d**) final crack morphology.

Figure 3, Figure 4 and Figure 5 show pictures of the cracking of specimens of different sizes. As shown in Figure 3, Figure 4, Figure 5 and Figure 6, the cracking process of the soil samples can be divided into four stages: initial stage, early development stage, late development stage, and final stabilization stage (fracture network). By monitoring the temperature and humidity of the soil samples in real time, it was found that the soil samples all started to sprout fissures at a temperature of 46 °C and a humidity of 95% (Figure 3b, Figure 4b, Figure 5b and Figure 6b). There was a rapid development of fissures in the soil samples as the experimentally set temperature changed (Figure 3c, Figure 4c, Figure 5c and Figure 6c). When the temperature was constant, the specimens eventually formed a fracture network and basically stopped cracking. The analysis of cracking images of soil samples with four aspect ratios showed that: ① soil samples with an aspect ratio of less than 3:1 (Figure 3d, Figure 4d and Figure 5d) had many secondary cracks, the final area of cracks was large, and the soil sample with a smaller aspect ratio the more complex the crack development path, the more severe the soil sample cracking; ② for soil samples with an aspect ratio greater than 6:1 (Figure 6d), the cracks appeared to penetrate up and down as the depth increased, accompanied by secondary cracks.

At the beginning of the experiment, under the action of capillary water pressure in-side the soil sample, the water continuously migrated to the surface layer [23]. Affected by the temperature difference between the internal and external environment of the soil sample, the surface water evaporated continuously, resulting in the formation of holes on the surface of the soil sample. With the progress of the experiment, the water in the lower part of the soil sample decreased and the water pressure decreased, resulting in an insufficient water supply on the surface of the upper soil sample, and the surface dehumidification rate was significantly accelerated. When the tensile stress was greater than the strength of the soil sample, cracks began to occur (Figure 3b). When the main fissure was formed, more secondary fissures with smaller widths were derived (Figure 3b, Figure 4b, Figure 5b and Figure 6b), and finally a fissure network was formed [16,17,18,19]. For soil samples with an aspect ratio of less than 3:1, for example, there were many secondary cracks in Figure 3d, Figure 4d, and Figure 5d, and for soil samples with an aspect ratio greater than 6:1, the cracks showed up and down penetration. The morphology had few secondary cracks (Figure 6d). This is because the specimen was relatively uniform, the aspect ratio of the specimen was 6:1, and the constraint of the wide side of the specimen was greater than that of the two ends, and the narrow length of the specimen will limit the crack development and cause the crack to penetrate up and down. Soil samples with small proportions generally generated the maximum stress in the middle due to the uniform restraint on all sides, and the obvious effect of stress led to more complicated soil sample cracks.

### 3.2. Crack Area Results Analysis

**Figure 7:**
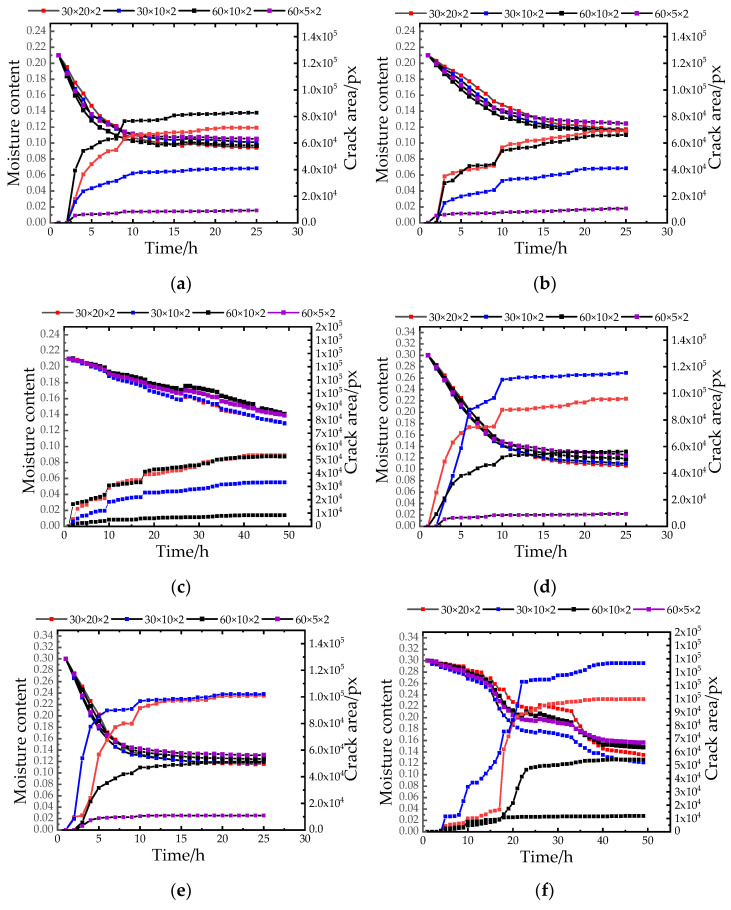

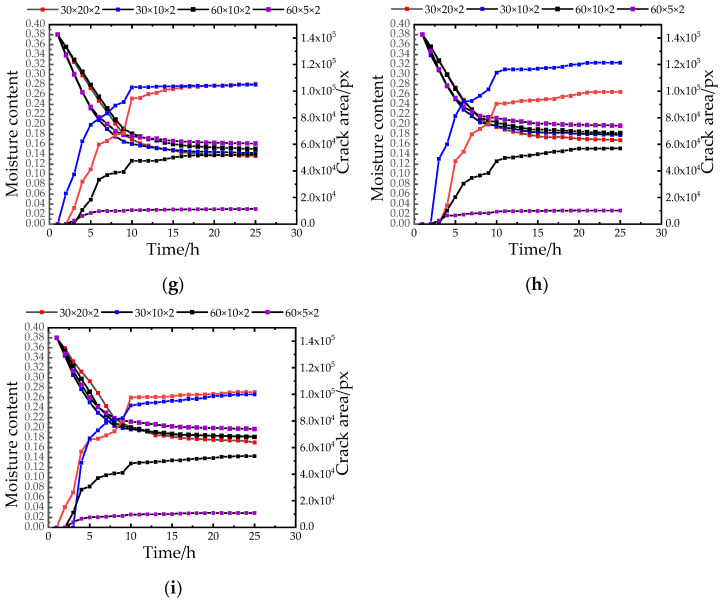
Fracture area curve. (**a**) the change curve of crack area under 21% initial moisture content and 30% humidity; (**b**) the crack area change curve under 21% initial moisture content and 67% humidity; (**c**) the change curve of crack area under 21% initial moisture content and 95% humidity; (**d**) the crack area change curve under 30% initial moisture content and 30% humidity; (**e**) the crack area change curve under 30% initial moisture content and 67% humidity; (**f**) the change curve of crack area under 30% initial moisture content and 95% humidity; (**g**) the variation curve of crack area under 38% initial moisture content and 30% humidity; (**h**) the change curve of crack area under 38% initial moisture content and 67% humidity; (**i**) the change curve of crack area under 38% initial moisture content and 95% humidity.

Figure 7 shows the relationship between water content and fracture area. From the results in Figure 7, it can be seen that with the transition of the temperature gradient, when the high temperature dropped to the next gradient low temperature, the water content at the temperature transition node decreased significantly, and then gradually became stable. From the second gradient to the third gradient, the water content decreased significantly again and then tended to be stable at the cooling node again. Therefore, the change in temperature had a great influence on soil moisture content and fissure area. At each temperature transition node, the fissure area increased. In the case of 0 h–2 h high temperature, due to the high temperature, the water in the environment was converted into water droplets and accumulated on the upper surface of the sample and penetrated into the soil sample, resulting in a small increase in the water content. As the experiment was carried out, the soil sample was formed with cracks, which provided a wider channel for the migration of moisture inside the soil sample, so the moisture content decreased, resulting in a substantial increase in the crack area [24,25]. Moreover, in the drying process, the evaporation of surface water led to tensile stress on the surface of the soil sample. When the tensile stress reached the tensile strength of the local soil sample, cracks were generated at the stress concentration.

The internal moisture content of the soil sample also has a certain impact on the crack area. The high moisture content soil sample lost water faster, the cracks appeared earlier, the number of cracks was more, and the crack was more likely to occur than the low moisture content soil sample. Because there was a lot of water in the soil sample with high water content, under the action of the environment and capillary water, the water migrated more, the water migrated to the surface of the sample quickly, the evaporation rate was fast, the water content of the soil sample dropped rapidly, and cracks were formed quickly. From the test results, it can be seen that the high moisture content soil samples began to have obvious cracks and developed rapidly in the early 1 h of the experiment. The internal moisture content of the soil samples with a low moisture content decreased gradually, the initiation and development of cracks was slow, and small cracks appeared only after 3–4 h.

Samples of different sizes were affected by different aspect ratios, and the rate of water evaporation, surface tension, degree of cracking, and laws were also different. Therefore, in the excavation process of the earth site, the aspect ratio of the excavation surface plays an important role in the protection of the earth site. As shown in Figure 7, under the influence of different temperature and humidity, the crack area of the samples with different aspect ratios increased rapidly at the initial stage of the experiment. When the length–width ratio of the soil sample with the initial moisture content was less than 3:1, the crack area rise rate was the fastest, and the final crack area was also the largest. With the decrease in humidity, the crack area of specimens with an aspect ratio of less than 3:1 (Figure 7a,b,d,e,g,h) increased slowly, and finally the crack area was the smallest, the crack area of the sample with a larger length-width ratio was smaller, and the crack of the sample with a similar length and width was severe.

### 3.3. Analysis of Crack Orientation Results

**Figure 8:**
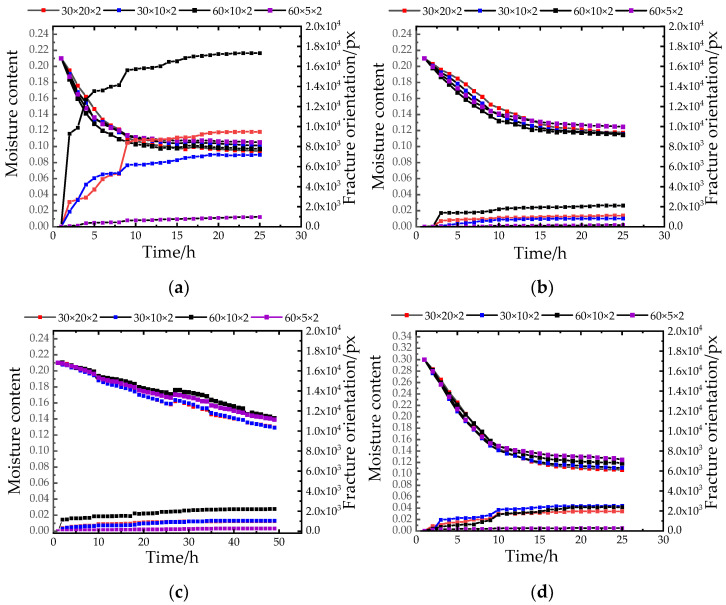

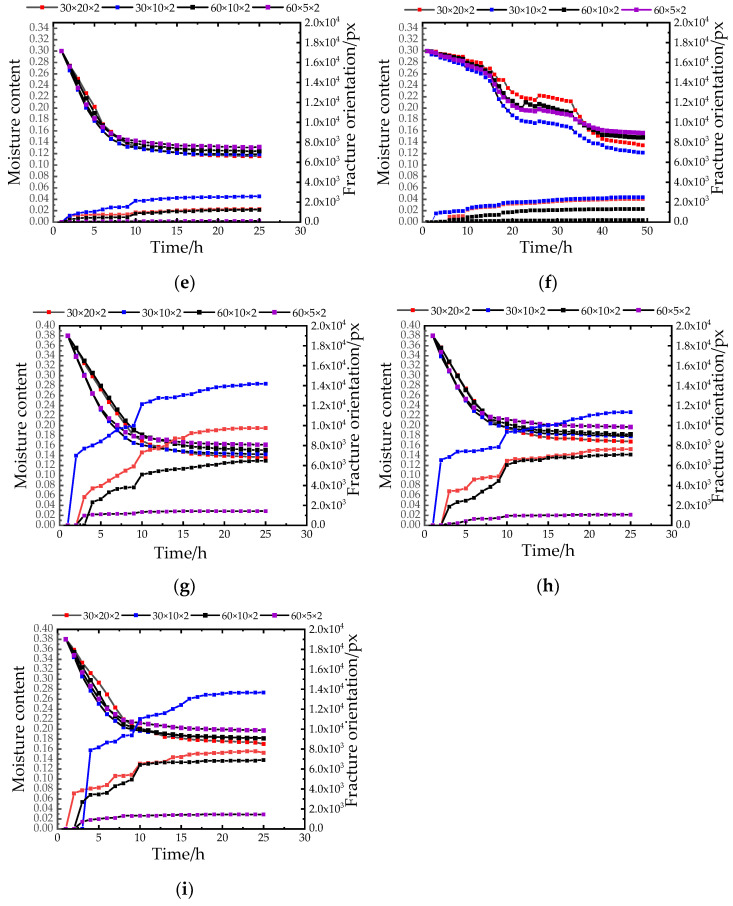
Crack orientation curve. (**a**) the change curve of crack orientation under 21% initial moisture content and 30% humidity; (**b**) the change curve of crack orientation under 21% initial moisture content and 67% humidity; (**c**) the change curve of crack orientation under 21% initial moisture content and 95% humidity; (**d**) the crack orientation change curve under 30% initial moisture content and 30% humidity; (**e**) the crack orientation change under 30% initial moisture content and 67% humidity; (**f**) the change curve of crack orientation under 30% initial moisture content and 95% humidity; (**g**) the crack change curve 38% initial moisture content and 30% humidity; (**h**) the change curve of crack orientation under 38% initial moisture content and 67% humidity; (**i**) the change curve of crack orientation under 38% initial moisture content and 95% humidity.

Figure 8 shows the relationship between moisture content and crack orientation. As shown in Figure 8, it can be seen that with the continuous change of the environment, the sample first formed a single main crack (primary crack), and as the experiment proceeded, it germinated on the basis of the primary crack. Secondary cracks and tertiary cracks were generated. It can be seen from the macroscopic cracking pictures that the development of cracks basically kept developing in the direction of the long side first, then secondary and tertiary secondary cracks were formed in the direction of the short side, and finally the cracks were opened in a crisscross pattern, and the two-level cracks were basically a Y. shape or 90°, eventually forming a network of fissures. The complexity of the cracks reflected the degree of damage to the sample. During the experiment, stress concentrations occurred at the weak part of the soil sample surface, resulting in the change of the crack orientation of the sample under the action of stress. When the experiment was carried out for 8 h, at the temperature gradient change node, the aspect ratio increased significantly, indicating that new cracks were generated at this stage and the crack development was obvious.

As shown in Figure 8c,f,i, compared with the low humidity conditions of 30% and 67%, the aspect ratio of the closed figure fluctuated less in high humidity conditions. Combined with the experimental samples, it can be seen that the sample developed cracks in a high humidity environment to form a closed pattern with a small area and a large crack area, indicating that the sample had many and small closed patterns, a complex development, and the transfer distribution of the stress field was disordered and varied. Therefore, under the condition of low humidity, the damage degree of the sample was less, and this kind of environment was more conducive to the protection of the site soil.

When the water content was 21% and the aspect ratio was greater than 6:1 (Figure 8a–c), the number of cracks was small, the crack development was stable, and the total aspect ratio of the crack closed pattern was relatively high. This is because the soil moisture content was low and the water loss was fast, the stress was relatively concentrated, and the stress field was relatively fixed. When the moisture content increased to 30% and the aspect ratio of 38% was less than 6:1 (Figure 8d–i), the length and width of the closed graph was larger than the sum, the number of cracks was large, and the number of closed figures was formed. This is because the stress field constantly changed with the test, resulting in the continuous transfer of stress concentration points, thus causing damage to many parts of the soil sample.

Due to the relatively uniform soil structure of the sample, the orderly concentration and release of strain energy was promoted, which in turn controlled the grade continuity characteristics of crack development. When the sample was dried to the residual moisture content, the development of the fracture network tended to stop. This test took the Kaifeng Zhouqiao site as the test object, and the test phenomenon was consistent with the site. For longitudinal penetrating cracks, the entire section was cracked in the vertical direction, increasing the possibility of its collapse and destroying the site; transverse cracks and longitudinal cracks interacted with each other. The intersection was intricate, and this situation existed at the site. After the crack occurred, the soil damage was deepened under the long-term external environment and salt migration and other factors.

### 3.4. Analysis of Crack Angle Results

**Figure 9:**
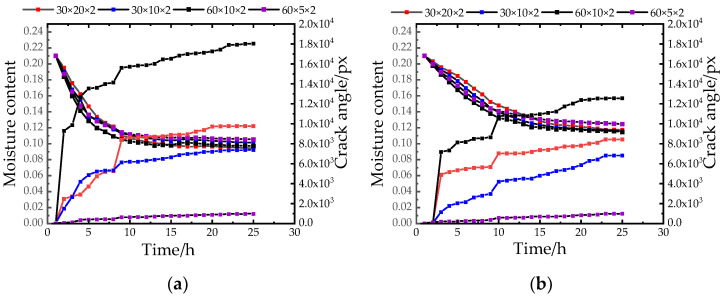

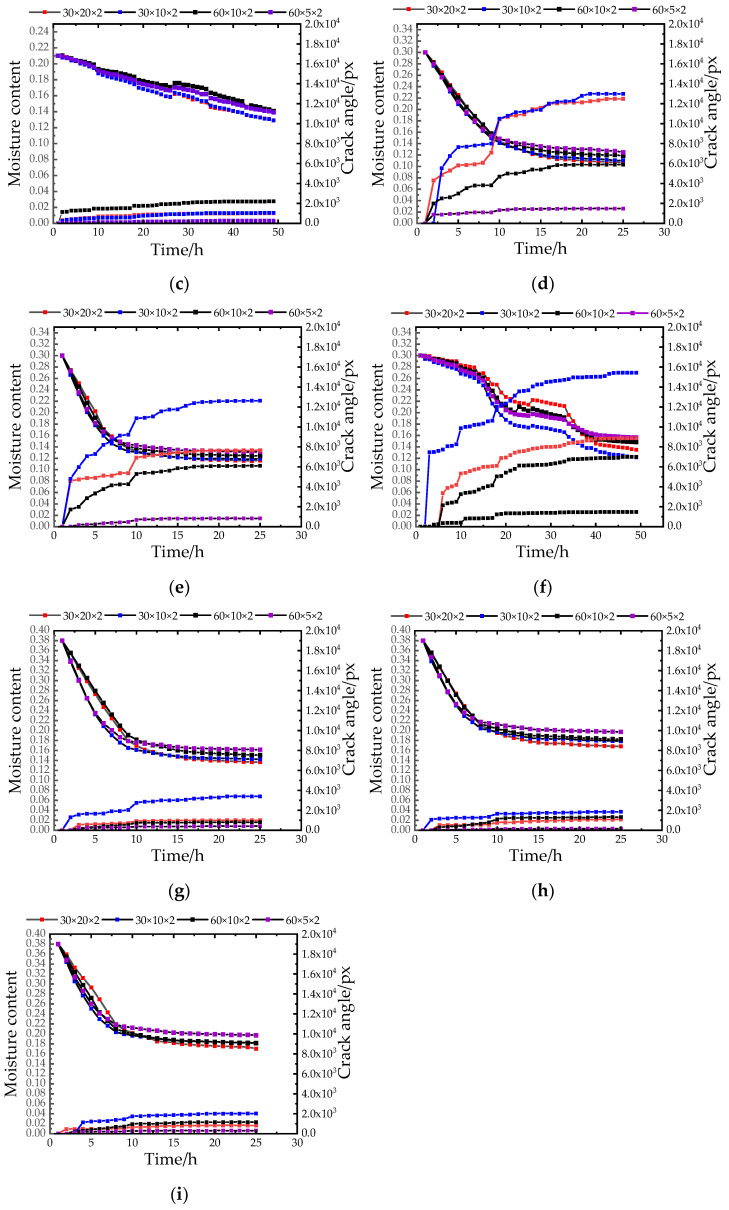
Crack angle change curve. (**a**) the change curve of crack angle under 21% initial moisture content and 30% humidity; (**b**) the change curve of crack angle under 21% initial moisture content and 67% humidity; (**c**) the change curve of crack angle under 21% initial moisture content and 95% humidity; (**d**) the crack angle change curve under 30% initial moisture content and 30% humidity, (**e**) the crack angle change under 30% initial moisture content and 67% humidity Curve; (**f**) the change curve of crack angle under 30% initial moisture content and 95% humidity; (**g**) the change curve of crack angle under 38% initial moisture content and 30% humidity; (**h**) the change curve of crack angle under 38% initial moisture content and 67% humidity; (**i**) the change curve of crack angle under 38% initial moisture content and 95% humidity.

Figure 9 shows the relationship between the moisture content and crack angle. As shown in Figure 9, it was caused by the boundary effect of soil samples [26,27,28,29]. All samples started to initiate and develop cracks at the contact with the mold. The development process of dry shrinkage cracks was the result of the combined action of two boundary conditions: the evaporation water loss of the upper interface and the friction of the lower interface. When the friction is small, the bottom displacement–limited fissure development is not obvious [30,31,32]. Considering the problem of boundary effect, the mold used in this experiment was made of smooth stainless steel, and the friction between it and the soil sample was small, so the cohesive force was small. Therefore, the cracking of the soil sample was dominated by surface evaporation and water loss. The phenomenon of local stress concentration redistributed the stress field and formed a new stress concentration at the tip of the crack, which promoted the continuous extension and expansion of the crack.

At the beginning of the experiment, the angle started from zero by default, and the crack angle began to change with the initiation of cracks. In the early stage of the experiment, the increase rate of the crack angle was fast, and as the experiment progressed, the increase rate of the crack angle slowed down until it stopped rising at the end. This is consistent with the development law of soil sample cracks. When the crack occurred, the soil sample shrank at the edge in contact with the mold and separated from the mold along the periphery of the mold. As the crack gradually expanded and extended to the middle of the sample, under the influence of stress redistribution, the stress concentration position changed, and it was found that the newly generated secondary and tertiary cracks were Y-shaped or 90° with the upper cracks (Figure 9b–d). Because the soil sample contained a lot of water in the early stage of the experiment, the continuous change of the stress field of the soil sample under the action of the environment led to the continuous development of cracks, resulting in the continuous change of the angle. Moreover, the crack angle was also no longer developed, so the crack angle did not change. The index of the crack development angle provides a reference for the study of crack development law, which is conducive to grasping the dynamic development law of cracks, and then provides a useful reference for the excavation of soil sites according to the law.

Under the same temperature and low humidity (30%) environment, the crack angle developed rapidly in the early stage of the experiment, and in the high humidity (95%) environment, the overall crack angle development showed a smooth and slow trend; however, for the samples in the intermediate humidity (67%) state, the total crack angle caused by drying shrinkage was small and the growth was slow. Therefore, for site protection, keeping the soil sample at an intermediate humidity is an ideal condition to reduce the development of the crack angle of the soil sample. For example, from Figure 9a it can be seen from the lateral comparison observation that when the aspect ratio was large, the soil samples with the initial moisture content of 21% and 38% (Figure 9a–f). Therefore, choosing the appropriate excavation aspect ratio hac a great influence on the development of the crack angle of the soil site.

## 4. Conclusions

During the excavation process of the soil site, the surface moisture caused shrinkage cracking caused by the change of moisture content due to environmental influence. This paper took the Zhouqiao soil site as the research object, based on the actual weather conditions and the excavation area of the site in Kaifeng City, through the control variable method. The influence of temperature and humidity, initial moisture content, mold sizem and other factors were analyzed through the drying shrinkage test. Through image capture and processing technology, PS and IPP software were used to binarize and denoise the image, and comprehensively explore the change law of the crack area, crack orientation, and crack angle of soil samples from the site under the condition of dry shrinkage. The main conclusions are as follows.

(1) The length–width ratio of soil samples has a great influence on the cracking law. The soil sample with an aspect ratio of less than 3:1 had more secondary cracks, and the crack area was large. For soil samples with an aspect ratio greater than 6:1, the cracks appeared to penetrate up and down as the depth increases.

(2) The soil sample with an aspect ratio of less than 3:1 had a faster crack area increase rate than the sample with an aspect ratio of more than 3:1, and the total crack area was the largest. The soil samples with a high moisture content lost water faster, and the cracks appeared earlier and the number of cracks was more than that of the soil samples with a low moisture content, which were more prone to cracking.

(3) The change law of crack orientation is consistent with the change law of water content. When the temperature is fixed, the high humidity environment is more likely to lead to soil cracking, complex development, indeterminate crack orientation, and chaotic crack network, while the damage degree of the sample under a low humidity is less. Therefore, conditions of low humidity are more conducive to the preservation of the site soil. 

(4) The transformation law of the crack angle is consistent with the change law of crack development. The development of the crack angle of the sample was mainly concentrated in the early stage of the experiment, showing a Y-shaped or 90° development trend. With the progress of the experiment, the change of temperature and humidity had little effect on the change of the crack angle. When the aspect ratio of the sample was greater than 6:1, the sum of the crack angles was large, the number of cracks was large, and the cracks were intricate and honeycomb shaped. When the ratio was less than 6:1, the number of cracks was small but there were more deep cracks.

## Figures and Tables

**Table 1:** Basic parameters of the soil at the Zhouqiao site.

Liquid Limit/%	Plastic Limit/%	Plasticity Index	Max. Dry Density/(g/cm^3^)	Natural Water Content/%
38	21	17	1.519	12.5

**Table 2:** Dry shrinkage test scheme.

Test Number	Control Variables			Test Piece Combination
	Temperature (A)	Humidity (B)	Water Content (C)	
1	13 °C	30%	23%	A1B1C1
2	13 °C	67%	31%	A1B2C2
3	13 °C	95%	38%	A1B3C3
4	27 °C	30%	23%	A2B1C1
5	27 °C	67%	31%	A2B2C2
6	27 °C	95%	38%	A2B3C3
7	46 °C	30%	23%	A3B1C1
8	46 °C	67%	31%	A3B2C2
9	46 °C	95%	38%	A3B3C3

**Table 3:** Variance of crack area in the parallel test.

Group	a	b	c	d	e	f	g	h	i
Variance of fracture area	0.035	0.048	0.041	0.038	0.042	0.044	0.039	0.041	0.043

**Table 4:** Parallel test fracture azimuth variance.

Group	a	b	c	d	e	f	g	h	i
Fracture azimuth variance	0.036	0.047	0.042	0.046	0.039	0.043	0.037	0.041	0.038

**Table 5:** Parallel test crack angle variance.

Group	a	b	c	d	e	f	g	h	i
Fracture azimuth variance	0.037	0.042	0.042	0.049	0.037	0.043	0.037	0.041	0.038
